# Synoptic Operative Reporting in Cervical Cancer Surgeries: Experience From a Single Oncology Center

**DOI:** 10.7759/cureus.50462

**Published:** 2023-12-13

**Authors:** Nilanchali Singh, Michelle Chanco, Vivian Wen, Neha Mishra, Shivangi Mangal, Prafull Ghatage

**Affiliations:** 1 Obstetrics and Gynaecology, All India Institute of Medical Sciences, New Delhi, Delhi, IND; 2 Cancer Center, Alberta Health Services, Calgary, CAN; 3 Obstetrics and Gynaecology, Goverment Institute of Medical Sciences, Greater Noida, IND; 4 Obstetrics and Gynaecology, Atal Bihari Vajpayee Institute of Medical Sciences & Dr. Ram Manohar Lohia Hospital, Delhi, IND; 5 Gynecologic Oncology, Tom Baker Cancer Center, Calgary, CAN

**Keywords:** standardized reporting, resident training, narrative operative report, oncology practice, cervical cancer, synoptic operative report

## Abstract

Objective

In today’s era of highly methodological oncological practices in place, we have a huge database to regulate, and it is foreseeable that a humongous load of information is ahead of us that we need to organize and comprehend. With the advancement in surgical equipment and evolving procedures, we need to store the information in a transferrable, understandable, and systematic way to prevent any ebb in the future. The systematic recording of operative data is even more important for patient management, training, and research. Standardized reporting also helps surgical residents have a better understanding of all aspects of the procedure. This study aims to analyze the synoptic operative reporting in cervical cancer patients from December 2009 to February 2020 in a single tertiary care center dedicated to providing oncology services to patients. This study will analyze the understandability, volume, and ease of transference of data during the given time period.

Methodology

The Alberta Cancer Registry was contacted to obtain data from the synoptic operative reports. Synoptic Operative Reports of all the patients operated on cervical cancer from December 2009 to February 2020.

Results

The data were obtained for 574 patients. As many as 463 patients were operated on for stage 1 and 2 cervical cancers and 10 patients for advanced and recurrent cervical cancer. A total of 101 patients were operated on for high-grade cervical dysplasia (HSIL). Adenocarcinoma was the most common histology. Laparotomy was performed in 308 patients, whereas others had laparoscopic procedures. Details of the surgery from the beginning of the incision to closure were recorded. The cervical cancer template consisted of 356 questions. There were separate templates for advanced and early-stage cancer. However, even with the meticulously detailed report, an average of only eight minutes was taken by each user to complete the template.

Conclusion

The computerized synoptic operative report has an upper hand over the dictated documentation report along with the ease of execution without missing essential substance. Its utility as an educational tool is very promising. Therefore, we encourage other facilities, especially cancer centers, to use synoptic operative reports more extensively not only for cervical cancer surgeries but also for other ones.

## Introduction

In the present scenario, documentation has become a powerful tool for medical professionals, especially in surgical fields. Among all the medical documents available, the surgical operative report can be considered the most important. A synoptic operative report (SOR) is a summarized document containing predefined leading criteria of the surgical procedure that can be conveniently completed in computerized templates [1]. The most common way of reporting used in hospitals worldwide is the narrative operative report (NOR), which is perceived as effortless but has its own derelictions. A handwritten report is amenable to missing substantial information, and the content of it depends on the virtue of the doctor who writes the note [2]. For a long time, the NOR has been used; however, it depends on the person's perspective rather than the factual report [3]. Therefore, a proper format for surgical reporting is an irreplaceable need. Evidence suggests that the 19-item WHO surgical safety checklist has helped minimize errors and adverse events and strengthen teamwork and communication [4].

Similarly, recording surgical data in a systematic way enhances its utility, keeps a check on any compromise on patient safety, improves resident training, and paves the way for future research. With standardized and comprehensive reporting of surgical events, surgical residents have a better understanding of all the facets of the procedure. With repeated systemic entry, there is a reinforcement of correct surgical steps among the surgeons and trainees. This study aims to analyze the report of cervical cancer by SOR from a single oncology center in the province of Alberta, Canada.

This article was previously presented as a free paper at the 64th All India Congress of Obstetrics & Gynaecology Annual Conference in 2021.

## Materials and methods

Study design, setting, and data resources

Comprehensive cancer care is provided through 17 oncology centers of Alberta Health Services to a population of more than four million residents in Alberta, Canada. It was a population-based retrospective study conducted at the Tom Baker Cancer Center (TBCC), Calgary, Alberta. There are two academic institutions in the province, and the Tom Baker Cancer Center is one of them. The Alberta Cancer Registry (ACR) is a population-based registry that prospectively collects data on patient demographics, tumor characteristics, and treatment details of patients diagnosed and treated in the province. The operative details are recorded through software called SORs. This system was designed and introduced in TBCC in 2009. Since then, the operative data of all operated cases of gynecological cancers have been recorded via synoptic operative reports.

Study population

Patients with cervical cancer operated on at the TBCC were included in the study. ACR was contacted to obtain data from the synoptic operative reports of these patients from December 2009 (when synoptic reporting of cervical cancer was started) to February 2020. The Alberta Cancer Committee Health Research Ethics Board approved the study before data collection. After reviewing the data from the software, 473 cervical cancer patients were operated upon during the given time. Thus, data from all 473 patients were compiled. The study design, analysis, and results are described in accordance with the Strengthening the Reporting of Observational Studies in Epidemiology (STROBE) guidelines [5]. Biomedical research was observational. The data were entered in an MS Excel sheet, and the results were reported in percentages.

## Results

Patient characteristics

Data were collected from 473 patients, of which 463 patients were operated on for Stage 1 and 2 cervical cancers and 10 patients for advanced and recurrent cervical cancer. The largest group of patients belonged to Stage IB1 (i.e., 239 patients, 51.6%), followed by the high-grade cervical dysplasia (HSIL) group (i.e., 101, 21.8%). On histological examination of the specimen, 188 (40.6%) were found to have squamous cell carcinoma, 125 (27%) belonged to the HSIL group, while adenocarcinoma was reported in 118 (25.49%) patients. Positive peritoneal cytology was found in 204 (44.06%) patients, while negative in 165 (35.4%) patients. Peritoneal cytology was not performed in 94 patients. Regarding the surgical approach, laparotomy was the first choice in most patients (66.3%), while laparoscopy was performed in 139 (30.02%) patients. Laparoscopy, followed by laparotomy, was performed in 13 (2.8%) patients, and a vaginal approach was followed in only four cases. Pelvic lymph node sampling was performed in 461 (99.57 %) patients, aortic lymph node sampling was performed in 13 (2.8 %), and appendectomy was performed in five patients (Table 1).

**Table 1:** Characteristics of the patient The data have been represented as N (number) and % (percentages of the total value).

Patient Details		N=463
Mean Age (years) (range)		46.3 (22.2 -91.3 years)
Histopathology, n(%)	Adenocarcinoma	118 (25.49%)
	Adenosquamous	7 (1.51 %)
	Carcinoma in situ	125 (27.00 %)
	Other	25 (5.40 %)
	Squamous	188 (40.6%)
Peritoneal Cytology, n(%)	Not done	94 (20.30%)
	No	165 (35.64%)
	Yes	204 (44.06% )
Surgical Approach, n(%)	Laparoscopy	139 (30.02%)
	Laparoscopy Followed by Laparotomy	13 (2.81%)
	Laparotomy	307 (66.31%)
	Vaginal	4 (0.86%)
Pelvic Node Sampling, n(%)	Not Done	2 (0.43 %)
	Done	461 (99.57%)
Aortic Lymph Node Dissection, n(%)	Not Done	450 (97.2 %)
	Done	13 (2.8 %)
Appendectomy, n(%)	Not Done	458 (98.9%)
	Done	5 (1.1%)

Types of surgeries performed in different stages of a carcinoma cervix

Laparotomy was the first-choice surgery in all stages of a carcinoma cervix. Of the total of 80 patients in Stage IA1, 43(53%) underwent laparotomy, and 34(42%) underwent laparoscopy. Laparoscopic-assisted surgery was performed in one case, while vaginal surgery was performed in two patients. A total of 18 patients belonged to Stage IA2, of which 13 (72%) underwent laparotomy, while laparoscopic surgery was performed in five (28%) of the patients. In Stage IB1, 150 (63%) underwent laparotomy, and 75 (31%) had laparoscopic surgery. Laparoscopy-assisted and vaginal procedures were performed in 12 (5%) and two (1%) of the patients, respectively. For 25 patients belonging to Stage IB2, laparotomy was performed in 22 patients, and laparoscopy in three patients. Overall, assisted laparoscopic and vaginal surgeries were rare (Table 2).

**Table 2:** Types of surgeries performed in different stages of a carcinoma cervix The data have been represented as N (number) and % (percentages of the total value)

Stage	Laparoscopic	Laparoscopy Assisted	Laparotomy	Vaginal	Total
IA1	34 (42.5%)	1 (1.25%)	43 (53.75%)	2 (2.5%)	80
IA2	5 (27.7%)	0	13 (72.2%)	0	18
IB1	75 (31.38%)	12 (5.02%)	150 (62.76%)	2 (0.8%)	239
IB2	3 (12.0%)	0	22 (88.0%)	0	25
HSIL	22 (21.78%)	0	79 (78.22%)	0	101

Details provided by SORs

The cervical cancer template consisted of 356 questions. There were separate templates for early-stage and advanced cancer. Despite providing such meticulous details, on average, an average of eight minutes were taken by each user to fill all the templates (Table 3).

**Table 3:** Example of the difference between narrative and synoptic reporting

Narrative Reporting	Synoptic Reporting
Abdomen was opened. Hysterectomy performed. Bilaternal salpingo-oophorectomy performed. Right pelvic lymphadenectomy done. Left pelvic lymphadenectomy not performed. Omentectomy done. Para-aortic lymphadenectomy performed. Intra-abdominal drain put. Hemostasis ensured.	Surgical Incision: Midline
	Laparotomy hysterectomy: Yes
	Right salpingectomy: Yes
	Left salpingectomy: Yes
	Left oophorectomy: Yes
	Right oophorectomy: Yes
	Rt obturator nodes: Yes
	Rt pelvic nodes: Yes
	Left obturator nodes: No
	Left pelvic nodes: No
	Right Ureterolysis: Yes
	Left ureterolysis: Yes
	Supracolic omentectmoy: No
	Infracolic omentectomy: Yes
	Para-aortic Lympadenectomy upto IMA: Yes
	Para-aortic Lympadenectomy upto Lt renal A: No
	Drain: Yes
	Type of drain: JacksonPratt
	Drain location: Left lower quadrant
	Hemostatic agent used: Surgicel

SOR software provides exhaustive details about the patient, including preoperative status, intraoperative procedure, and postoperative follow-up. Patient details start with age, ethnicity, body mass index, and previous surgical history. The preoperative details consisted of the American Society of Anaesthesiologists (ASA) score, Eastern Cooperative Oncology Group (ECOG) performance status, Karnofsky performance status, staging, metastasis (if any), primary/recurrent cancer, preoperative investigations, tumor markers, radiological findings, previous radiation therapy, and chemotherapy details. The intraoperative details provided were incision type, presence of ascites, cytology performed or not, detailed stepwise description of the disease, extent of surgical dissection, lymph node dissection, systematic details about surgery, suture used, closure, blood loss, blood replacement, and operative time. The postoperative details were also very meticulous, including the use of anticoagulation and follow-up plans, etc. (Table 4).

**Table 4:** Overview of the details entered in the synoptic operative reports *ASA - American Society of Anaesthesiologists #ECOG - Eastern Cooperative Oncology Group $BMI - Body Mass Index

Patient Details	Preoperative Details	Intraoperative Details	Postoperative Details
Age	ASA score^*^	Incision type	Status of patient
Ethnicity	ECOG performance status^#^	Catheterization	Transfer details
BMI^$^	Karnofsky performance status	Cytology done/not done	Tumor banking
Menopausal status	Stage	Ascites present	Anticoagulation
Weight change	Metastatic/local spread	Visible disease in abdomen and pelvis (detailed stepwise)	Specific comment
Symptoms	Primary/recurrent	Approach	Area for addendum
History of abnormal screening/cytology	Pre-op investigations (chest X-ray)	Extent of dissection	Follow-up plans
Last Pap smear	Tumor markers	Lymph node dissection	Time of follow-up
Contraceptive use	Radiological findings	Surgical details (systematic)	Person following up in the post-operative period
Family history	Histology	Closure	
History of surgery with details	Preoperative radiotherapy details	Suture details	
	Specific comment from the preoperative period	Blood loss	
	Previous chemotherapy	Blood replacement	
	Type of chemotherapy	Operative time	
	Comment on chemotherapy		
	Interval from preoperative treatment		
	Delay in surgery		
	Reason for delay		
	Antibiotic with dose		
	Anticoagulation received		

## Discussion

Approximately 234.2 million major surgical procedures take place worldwide in a year [6]. The Good Surgical Practice of the Royal College of Surgeons states the importance of safety and quality of surgical procedures, as well as optimal teaching and building of knowledge in the interest of the patient [7]. A major arrangement to do this is to refine the documentation of the main procedures. The 1992 report of the National Confidential Enquiry into Perioperative Deaths (NCEPOD) noted a considerable variation in the quality of the operation notes submitted by all contributing surgical specialties [8]. An audit of operative notes written by consultants and junior residents found that postoperative instructions were absent in almost two-thirds of operation notes, and serial numbers of prostheses were rarely recorded. Trainee doctors performed better than consultants, and emergency surgical notes were better than elective procedural notes [9]. The audit also gave a significant conclusion that 70% of the notes were illegible. It started in 2011 when many reviews done comparing SORs and NORs have shown that, in terms of overall completeness, completeness of subsections, and user-friendliness, SORs are significantly better than NORs [1]. As depicted in our study, synoptic operative report software includes exhaustive details of preoperative, intraoperative, and postoperative parameters. Miniscule but fundamental parts of the surgery might be missed in narrative reports including, but not restricted to missing important dates, complications during surgery, and the preoperative and postoperative procedures. On the other hand, synoptic reporting is a bridge between accuracy and consistency of entered diagnostic and prognostic information, and it reduces errors such as typography, transcription errors, and specimen turn-around time substantially. It helps in the exchange of data into other databases and better communication, thus facilitating research work. Not only there is ease in filling up already uploaded templates, but there is also ease in the transference of the information, standardization of the procedure, increased accuracy, administrative comfort in storing data, retrieval of data, prevention of any loss of paperwork, and formulation of new practices based on those notes and educative to trainees [10].

The same has been confirmed by our study that the synoptic software template helped capture most of the data about operated cervical cancer patients in the shortest time. This format may be a useful teaching tool for residents and trainees who can develop reporting skills if trained by synoptic reporting techniques. It ensures a uniform pattern, regardless of the person completing it. When Gur et al. compared 60 consecutive narrative breast cancer surgical reports and computerized synoptic reports in a prospective study over 10 months, they found that the SOR had 94.7% preoperative and operative data, while the dictated operative report had just 66% data (P<0.001) [10].

We observed that it only took eight minutes to complete the SOR for cervical cancer patients at our institute. Edhemovic et al. studied a computerized synoptic operative report template (WebSMR) in rectal cancer surgery in comparison to the standard operative narrative record used previously from 2001 to 2003 in seven hospitals in southern Alberta. The synoptic template consisted of all the data on the procedure, demographics, diagnostic evaluation, staging, and functional measures. They concluded that the surgical technique could be evaluated better with WebSMR, and it took only six minutes to fill it [11]. In their systematic review, Eryigit et al. found that synoptic reporting was linked with a higher completion rate in much less time, except for specific details about the operating method, which was more elaborate in a narrative report. Data extraction time was significantly shorter with synoptic software [1]. In a study to investigate the totality of the SOR compared to the dictated operative report for laparoscopic cholecystectomy and pancreaticoduodenectomy in six institutions, it was stated that the SOR was more comprehensive, and the majority of surgeons preferred it [12].

SORs are self-reflective, and if a surgeon has missed a step, then it is easy to figure out and correct their mistakes. The WHO 19-pointer surgical safety checklist is an example of how a pre-formed template, adherence to the steps, and effective communication can decrease complication rates [13]. SORs are not only meaningful in surgical notes, but the College of American Pathologists has been advocating standardizing the reporting of cancers for the past 20 years after the Pathology and Laboratory Medicine published that pathologists should begin to use checklists in their cancer reports [14,15]. It also came from the need for detailed reporting of tumors, and synoptic reporting was a measure to make it simpler and comprehensive. It was done not only to improve cancer registries and tumor staging reporting but also to improve long-term healthcare planning and create new knowledge [16]. Similarly, in surgical oncology, mentioning important structures such as lymph node involvement, peritoneal, and omental involvement, that is, mentioning the staging of cancer, is vital for the completeness of the follow-up treatment. This vital information is preserved when we use standardized templates even if new residents write the notes. For smaller procedures, it also helps fill a small timeless set of repetitive data. It also serves as a structured database for clinicians and policymakers. There are barriers that come along with it. There is a need to revise the software every time there is any advancement or change of procedure, sharing the information in the same software across hospitals and states, along with the professional resistance that comes with it. Information overload, multiple templates that may surpass human cognition, and software complicacies that may cause hindrance, along with unavailability in third-world countries, are a few obstacles. Information overload in the synoptic reporting presentation layer can lead users to ignore, overlook, or misinterpret crucial information.

SORs are now being used in various fields of medicine, including radiology, internal medicine, oncology, oncopathology, trauma, and gastosurgery. At the TBCC in Alberta, Canada, a synoptic surgical template was created for breast cancer surgery and had an acceptance rate of 89%, and 50% of surgeons could finish it in five minutes, showing that a good template, if made, can be easily implemented in the system [17]. Operative notes have been found to not achieve their fundamental goals and have difficulty in extracting valuable and interpretable data [18,19]. A study was conducted to see the comparison between narrative and synoptic reporting of a very common procedure such as laparoscopic cystectomy by analyzing the operative videos and notes. It showed that narrative notes were not exactly reflective of the surgical steps, and a few complications were deliberately omitted [3]. This has led many institutes to implement synoptic reporting of surgical notes as the standard method of documentation and has improved surgical outcomes and medical care provided, by increasing compliance with surgical safety checklists and has improved feedback and reporting [20]. In conclusion, the computerized method of reporting has many credits to optimization to data storage and ease of execution; hospitals can build up their own software to be used universally in the states for transference. It will serve as a boon for medical and surgical education and will improve patient safety.

## Conclusions

The present study found that synoptic operative reporting captures the maximum amount of data with a clear understanding in an average of eight minutes with regard to cervical cancer surgeries. Hence, this study inferred that it is crucial to capture surgical data precisely, easing data exchange and analysis, guaranteeing quality control, facilitating the study of cancer research, the study of its epidemiology, and teaching residents. The synoptic operative report has an upper hand over the dictated documentation report along with the ease of execution without missing essential substance.

The utility of synoptic operative reporting as an educational tool is very promising. Therefore, we encourage other facilities, especially cancer centers, to use synoptic operative reports more extensively not only for cervical cancer surgeries but also for other ones. In the future, it may help share data among hospitals in a standardized manner and has a promising role to play in regularising patient safety and care by generalizing procedures.
